# The role of suprascapular nerve block in hydrodilatation for frozen shoulder

**DOI:** 10.1051/sicotj/2022026

**Published:** 2022-06-14

**Authors:** Rifki Albana, Renaldi Prasetia, Andri Primadhi, Agus Hadian Rahim, Yoyos Dias Ismiarto, Hermawan Nagar Rasyid

**Affiliations:** Department of Orthopaedics-Traumatology, Faculty of Medicine, Universitas Padjadjaran, Hasan Sadikin General Hospital 40161 Bandung Indonesia

**Keywords:** Frozen shoulder, Hydrodilatation, Corticosteroid, Suprascapular nerve block, Spinoglenoid notch

## Abstract

*Introduction*: Frozen shoulder is a debilitating problem that requires comprehensive diagnosis and management. Patients usually recover, but the possibility of not reobtaining a full range of motion exists. Thus, early shoulder exercises are necessary to achieve their full range of motion. This study aims to understand the effects of suprascapular nerve block (SSNB) augmentation at the spinoglenoid notch in hydrodilatation to treat frozen shoulder to facilitate early shoulder exercises. *Methods*: The current study retrospectively observed 31 patients, including 40–60-year-old patients diagnosed and treated with primary frozen shoulder. The participants were divided into groups A (hydrodilatation) and B (hydrodilatation and the augmentation of an SSNB). Shoulder function and pain scores were assessed before, during, and after the intervention (at months 1 and 6). *Results*: The result of this study shows that suprascapular nerve block plays a role in decreasing pain in intraintervention (0.69 vs. 5.73; *p* < 0.05), month 1 of follow-up (3.44 vs. 6.40; *p* < 0.05), but not significant on month 6 of group A and B after intervention (5.88 vs. 7.20; *p* > 0.05). Better delta functional scores were noted in the therapy group during month 1 of the follow-up (delta American shoulder and elbow surgeons [ASES]: 19.29 vs. 34.40, *p* < 0.05; delta disabilities of the arm, shoulder, and hand [DASH]: 17.88 vs. 38.15, *p* < 0.05). The difference in functional score on month 6 between both groups was not significantly different (delta ASES: 31.97 vs. 30.31, *p* > 0.05; delta DASH: 36.63 vs. 38.92, *p* > 0.05). *Discussion*: One rationale for using an SSNB augmentation at spinoglenoid notch in hydrodilatation for treating frozen shoulder was to obtain pain relief immediately to facilitate early manual exercise. SSNB has positive effects on short-term evaluation of shoulder pain and function after glenohumeral hydrodilatation, but not in the long term.

## Introduction

Frozen shoulder or adhesive capsulitis occurs in 3–5% of adults. The peak incidence of the cases is in individuals 40–60 years old [1]. The exact etiology of frozen shoulder remains unknown. Chronic inflammation and fibrosis are hypothesized to play a part in the pathogenesis of frozen shoulder [2].

Patients with frozen shoulder may suffer discomfort or even disability on the affected side of the shoulder, and the illness resolution may take months [3]. Several treatment modalities alleviate the reduced range of motion, e.g., rest, analgesia, active/passive mobilization, acupuncture, physiotherapy, oral/injected corticosteroid, capsule distention, and surgical capsule release [1]. However, no consensus exists on the most efficacious treatments for this condition [4].

Hydrodilatation of the shoulder joint capsule was a novel treatment to alleviate the pain of the affected shoulder. The procedure may be given with or without an adjuvant corticosteroid. Hydrodilatation, using saline and a corticosteroid, was superior in short-term pain reduction and range of motion improvement compared to management using only physiotherapy and corticosteroid injection in patients treated for frozen shoulder [5]. The proposed hypothesis regarding the mechanism of action in hydrodilatation and pain reduction in patients with the frozen shoulder was associated with synovitis and fibrosis removal [6, 7]. Previous studies also noted better pain reduction in patients receiving hydrodilatation with adjuvant corticosteroid than only corticosteroid injection [8, 9]. Several other adjuvants were studied in frozen shoulder treatment using hydrodilatation [5, 10].

The hydrodilatation method can cause pain in the shoulder during the intervention, so early manual exercise after the intervention is required to inhibit pain [11]. A suprascapular nerve block in the spinoglenoid notch provides pain relief during the intervention [1]. The choice of suprascapular nerve block is because the suprascapular, which arises from the C4–6 spinal nerves, branches out of the upper trunk of the brachial plexus and innervates 70% of the shoulder joint. Intervention in the spinoglenoid notch is expected to protect only the sensory and motor functions of the suprascapular nerve [7, 12–14]. The literature comparing the efficacy of suprascapular nerve block at the spinoglenoid notch used as an adjunct in hydrodilatation of frozen shoulder remains sparse, specifically in Indonesia.

Therefore, this study aims to understand the effects of augmentation of a suprascapular nerve block at the spinoglenoid notch in hydrodilatation to treat frozen shoulder hydrodilatation.

## Material and methods

### Study design

This study retrospectively observed the medical records of 31 patients (40–60 years old) diagnosed with and treated for primary frozen shoulder in freezing to the frozen phase transition, in Hasan Sadikin General Hospital, between 2019 and 2021.

### Inclusion criteria

This study included patients 40–60 years old, in the freezing to the frozen phase transition, included in the Zuckerman diagnostic criteria, and patients diagnosed with primary frozen shoulder.

Zuckerman’s criteria, mentioned in the inclusion criteria, consist of:

1. Sudden onset.
2. Range of motion (ROM) during the active and passive elevation of the shoulders <100°.
3. ROM limitation in external rotation <50% on the contralateral side.
4. Shoulder pain during the night.
5. Normal radiographical findings.

Exclusion criteria included other shoulder pathologies and some medication histories.

### Exclusion criteria

Patients who refuse the intervention; who have a history of shoulder surgery, breast surgery, and fracture around the shoulder and upper extremities; have a cerebrovascular disease and cervical radiculopathy; with systemic disease (e.g., diabetes mellitus); who underwent shoulder surgery during the study; who obtained other additional therapy outside the study; and have secondary and tertiary frozen shoulder including extracapsular pathology were excluded from this study.

### Sample collection

The samples in the study were divided into two groups. Group A received hydrodilatation with saline and corticosteroid injection without suprascapular nerve block augmentation at the spinoglenoid notch. Group B received hydrodilatation with saline, corticosteroid injection, and suprascapular nerve block augmentation at the spinoglenoid notch. All samples were conducted with three types of examinations before the intervention, consisting of visual analog scale (VAS), American shoulder and elbow surgeons (ASES) scoring, and disabilities of the arm, shoulder, and hand (DASH) score.

### Intervention

Patients in both groups will receive hydrodilatation and corticosteroid injection under ultrasonography (USG) guidance. The posterior approach was used in all procedures. During the process, the operator stands behind the patient and monitors to accomplish an ergonomic position in line with the ultrasound. The ultrasound monitor is placed in front of the patient. The patient is in lateral decubitus with affected palm to nonaffected shoulder with padding to protect the prominent area of the body.

**Figure 1:**
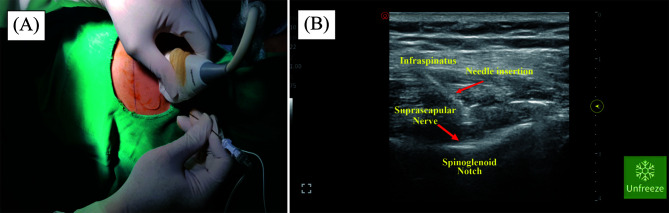
The suprascapular nerve at the supraglenoid notch using USG. The operator stands behind the patient, parallel to the ultrasound monitor, to achieve an ergonomic position. The ultrasound monitor is placed in front of the patient. The patient is in a lateral decubitus position, with the affected palm against the nonaffected shoulder and padding to protect the prominent area of the body. A sterile technique was used to prepare and clean the affected shoulder. The injection site for the suprascapular nerve block at the spinoglenoid notch is 2 cm below the spine scapula (A). An echogenic 23-G (3.5 in) spinal needle is inserted from medial to lateral (B). Injection (20 mg triamcinolone acetonide, 4 mL 2% lidocaine, and 4 mL 0.5% bupivacaine as a cocktail) was given and waited for 1–2 min.

**Figure 2:**
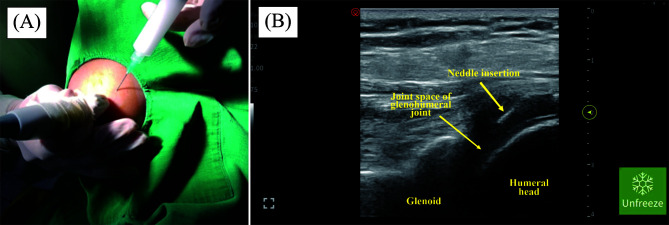
Identification and glenohumeral hydrodilatation on frozen shoulder. A posterior approach for glenohumeral joint injection created an anatomical landmark approximately 1–2 cm below the posterolateral acromion and shifted to the medial, short-axis probe position (A). Echogenic needle insertion in-plane position from the lateral to the medial, penetrating the infraspinatus muscle and posterior capsule, injecting the steroid (40 mg triamcinolone acetonide) as 15–20 mL aquabidest (B).

In group A, patients who received hydrodilatation used 15–20 mL of saline and 40 mg of triamcinolone and approached posteriorly for glenohumeral joint injection, starting 2 cm below the posterolateral acromion and shifting to the medial side (Figure 2). For group B, the patients received an augmentation suprascapular nerve block at the spinoglenoid notch with 4 mL bupivacaine (0.5%), 4 mL lidocaine (2%), and 20 mg triamcinolone before the hydrodilatation procedure. The injection site is 2 cm below the spine scapula (Figures 1 and 2).

### Clinical evaluation

Patients were regularly evaluated for VAS, ASES scoring, and DASH scores. The procedures and evaluation of all three scores were performed by the same physician in charge of the patient. The early manual exercise was prescribed after the hydrodilatation with or without suprascapular nerve block at the spinoglenoid notch. The first evaluation is VAS assessed in the intraintervention between both groups. The patients were also evaluated on month 1 of follow-up, and used VAS, DASH scoring, and ASES scoring at month 6 of follow-up.

### Data analysis

Data analyses were performed using the Statistical Program for Social Sciences, version 25. Univariate tests were performed using the *t*-test and Mann–Whitney test on intergroup comparisons. According to the previous normality test, paired *t*-tests and Wilcoxon tests were performed on each variable. A *P*-value of <0.05 was deemed significant. Normality tests were performed for the study before further analysis using the Shapiro–Wilk test.

## Results

**Figure 3:**
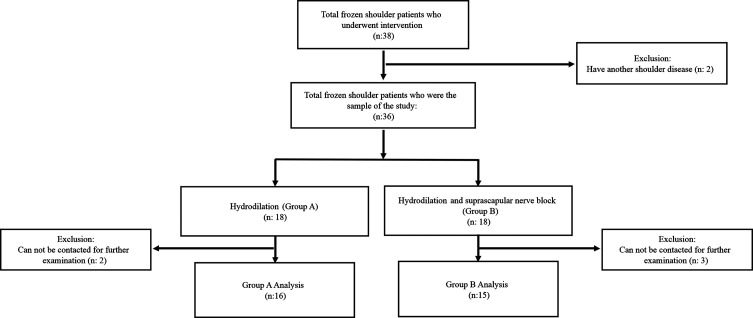
Research sampling flow. This study retrospectively observed and followed 31 patients. The samples were divided into two groups in the study. Group A received hydrodilatation with saline and corticosteroid injection without suprascapular nerve block augmentation at the spinoglenoid notch. Group B received hydrodilatation with saline, corticosteroid injection, and suprascapular nerve block augmentation at the spinoglenoid aperture. Groups A and B consisted of 16 and 15 patients, respectively.

Groups A and B consisted of 16 and 15 patients, respectively (Figure 3). No significant differences in baseline characteristics (Table 1) between both groups were noted (*p* > 0.05). In group A, the intrainjection VAS score was not significantly different from the preintervention score (*p* = 0.215), whereas group B had a significant difference (*p* < 0.001).

**Table 1:** Baseline characteristics.

Variables	Group A (*n* = 16)	Group B (*n* = 15)	*P*-value
Age (mean ± SD)	55.93 ± 13.18	52.81 ± 10.84	356
Sex			0.354
Male	3 (18.75%)	4 (26.67%)	
Female	13 (81.25%)	11 (73.3%)	
Affected side			0.853
Right	8 (50.0%)	7 (46.7%)	
Left	8 (50.0%)	8 (53.3%)	

Significant differences between VAS, ASES, and DASH scores were noted within the groups with pre- and postscores in all follow-up periods (Table 2; *p* = 0.001). Differences between groups A and B are outlined in Table 3. Significant differences exist in the delta between pre-and postscores between groups in several variables. Functional scores were significantly higher in month 1 in all three scores used for assessment (*p* < 0.05); however, no significant differences were noted in month 6 of reviews on all three scores (*p* > 0.05).

**Table 2:** Functional assessment for groups A and B before and after intervention.

Variables	Before	After	*P*-value
	Mean ± SD	Mean ± SD	
Group A			
VAS intra-injection	8.06 ± 1.28	7.38 ± 1.58	0.215*
VAS 1 month	8.06 ± 1.28	4.63 ± 1.14	0.000
VAS 6 month	8.06 ± 1.28	2.19 ± 0.83	0.000
ASES 1 month	24.34 ± 12.49	43.63 ± 12.52	0.000
ASES 6 month	24.34 ± 12.49	56.31 ± 17.88	0.000
DASH 1 month	59.01 ± 21.96	41.13 ± 18.34	0.000
DASH 6 month	59.01 ± 21.96	22.38 ± 14.052	0.000
Group B			
VAS intra-injection	7.87 ± 1.35	2.13 ± 0.35	0.001
VAS 1 month	7.87 ± 1.35	1.47 ± 1.12	0.001
VAS 6 month	7.87 ± 1.35	0.67 ± 0.90	0.001
ASES 1 month	27.75 ± 9.877	61.00 ± 14.74	0.000
ASES 6 month	27.75 ± 9.877	58.07 ± 16.48	0.000
DASH 1 month	49.56 ± 14.73	11.41 ± 11.25	0.001
DASH 6 month	49.56 ± 14.73	10.64 ± 9.01	0.001

Abbreviations: VAS, visual analog scale; ASES, American shoulder; DASH, disabilities of the arm, shoulder, and hand.

*Not significant result.

**Table 3:** Comparison of Delta scores before and after procedure between time periods.

Variables	Group A (*n* = 16)	Group B (*n* = 15)	*P*-value
VAS intra	0.69 ± 2.18	5.73 ± 1.33	0.000
VAS 1 month	3.44 ± 1.93	6.40 ± 2.09	0.000
VAS 6 month	5.88 ± 1.62	7.20 ± 2.00	0.052*
ASES 1 month	19.29 ± 13.19	34.40 ± 18.51	0.013
ASES 6 month	31.97 ± 18.78	30.31 ± 20.97	0.818*
DASH 1 month	17.88 ± 12.87	38.15 ± 15.27	0.000
DASH 6 month	36.63 ± 18.13	38.92 ± 15.11	0.707*

Abbreviations: VAS, visual analog scale; ASES, American shoulder; DASH, disabilities of the arm, shoulder, and hand.

*Not significant result.

## Discussion

Frozen shoulder is a debilitating problem that requires comprehensive diagnosis and management. Patients usually recover, but the possibility of not reobtaining a full ROM exists. Thus, early exercises are necessary to achieve their full ROM. The result of this study shows that suprascapular nerve block plays a role in decreasing pain in intraintervention (0.69 vs. 5.73; *p* < 0.05) and month 1 of the follow-up (3.44 vs. 6.40; *p* < 0.05), but not significant on month 6 group A and B after intervention (5.88 vs. 7.20; *p* > 0.05). Better delta functional scores were noted in the therapy group during month 1 of the follow-up (delta ASES: 19.29 vs. 34.40, *p* < 0.05; delta DASH: 17.88 vs. 38.15, *p* < 0.05). The difference in functional score on month 6 between both groups was not significantly different (delta ASES: 31.97 vs. 30.31, *p* > 0.05; delta DASH: 36.63 vs. 38.92, *p* > 0.05).

The study’s limitations were that this study used a retrospective study design and prospectively followed the development of patients’ pain and functional scores. The sample size and possible biases when selecting the patients were due to the single-center nature of the study. Bias may exist in the intervention results because the possibility of sample non-compliance with research procedures unknown to the researcher was noted. The self-limiting condition of the frozen shoulder meant that treatment should focus on restoring mobility and reducing pain in the affected shoulder to mitigate the possible impact on the quality of life.

Even though the frozen shoulder is a self-limiting condition, a study showed that the disease duration, if untreated, is around 4–36 months with an average of 15 months. Some studies show significant improvement in shoulder function, but other studies mentioned that 54% and 7% of patients had slight and marked restrictions, respectively [15]. The hydrodilatation method can provide mechanical adhesiolysis to facilitate increasing glenohumeral ROM. However, the procedure can create pain due to the overdistention of the glenohumeral joint capsule. The pain that follows will prevent early shoulder manual exercise from achieving functional shoulder ROM [11, 16–18]. One rationale for using a nerve block in frozen shoulder treatment was to give early pain relief from the affected shoulder [1, 19]. This study shows that suprascapular nerve block has decreased pain intervention. The suprascapular nerve block at the spinoglenoid notch performed in the study aimed to block the noxious stimuli to the glenohumeral joint from the suprascapular nerve. The suprascapular nerve innervated ~70% of the shoulder [13, 20]. Previous studies correlated earlier mobilizations with better outcomes in patients with frozen shoulders [17, 18].

The hydrodilatation of the shoulder capsule with saline and corticosteroids was the mainstay treatment for patients suffering from symptomatic frozen shoulders. Prior studies found significant improvement in function and pain reduction scores on the affected shoulder with a suprascapular nerve block [21]. Studies comparing the treatment with or without addition nerve block in hydrodilatation were relatively scarce.

Significant differences in pain and functional scores in this study were noted in both groups in the short-term evaluation. Therefore, the augmentation of suprascapular nerve block at the spinoglenoid notch in glenohumeral hydrodilatation for frozen shoulder remained effective. Better functional scores were noted in the therapy group during month 1 of the follow-up. In 2020, Majeed and Choukimath conducted a randomized controlled trial that mentioned that suprascapular nerve block improved faster and better than corticosteroid injections [21].

This study showed that the functional score in month 6 of follow-up between both groups was not significantly different. Previous studies noted that the suprascapular nerve block and intra-articular steroid injection only improved short-term outcomes (2 months after intervention) in terms of pain reduction and improvement in functional scores of the affected shoulder and not in long-term outcomes (1 year after the intervention), evaluated with function VASs (pain VAS and function VAS, respectively), ASES score, the Korean shoulder scoring system, the constant score, the simple shoulder test, and the shoulder pain and disability index [22]. Being a self-limiting condition, the treatment of frozen shoulder is meant to be symptomatic [23].

## Conclusion

A suprascapular nerve block at the spinoglenoid notch is an effective adjunctive treatment to hydrodilatation of shoulder capsules for frozen shoulder. The therapy effectively reduced pain and immediately improved functional scores to facilitate shoulder rehabilitation procedures to obtain an operating ROM.
